# Predicting Bone Adaptation in Astronauts during and after Spaceflight

**DOI:** 10.3390/life13112183

**Published:** 2023-11-09

**Authors:** Tannis D. Kemp, Bryce A. Besler, Leigh Gabel, Steven K. Boyd

**Affiliations:** 1 Department of Mechanical and Manufacturing Engineering, Schulich School of Engineering, University of Calgary, Calgary, AB T2N 1N4, Canada; 2 McCaig Institute for Bone and Joint Health, University of Calgary, Calgary, AB T2N 4Z6, Canada; 3 Department of Radiology, Cumming School of Medicine, University of Calgary, Calgary, AB T2N 4Z6, Canada

**Keywords:** bone remodeling, inverse problems, participant-specific prediction, level set methods, spaceflight, HR-pQCT

## Abstract

A method was previously developed to identify participant-specific parameters in a model of trabecular bone adaptation from longitudinal computed tomography (CT) imaging. In this study, we use these numerical methods to estimate changes in astronaut bone health during the distinct phases of spaceflight and recovery on Earth. Astronauts (N = 16) received high-resolution peripheral quantitative CT (HR-pQCT) scans of their distal tibia prior to launch (L), upon their return from an approximately six-month stay on the international space station (R+0), and after six (R+6) and 12 (R+12) months of recovery. To model trabecular bone adaptation, we determined participant-specific parameters at each time interval and estimated their bone structure at R+0, R+6, and R+12. To assess the fit of our model to this population, we compared static and dynamic bone morphometry as well as the Dice coefficient and symmetric distance at each measurement. In general, modeled and observed static morphometry were highly correlated (*R*^2^ *>* 0.94) and statistically different (*p* < 0.0001) but with errors close to HR-pQCT precision limits. Dynamic morphometry, which captures rates of bone adaptation, was poorly estimated by our model (*p* < 0.0001). The Dice coefficient and symmetric distance indicated a reasonable local fit between observed and predicted bone volumes. This work applies a general and versatile computational framework to test bone adaptation models. Future work can explore and test increasingly sophisticated models (e.g., those including load or physiological factors) on a participant-specific basis.

## 1. Introduction

During spaceflight, astronauts lose bone approximately 10 times quicker than with age-related bone loss [1,2], providing an accelerated timeline to study bone loss on Earth. Accelerated bone loss during spaceflight likely occurs due to a number of factors, including a lack of mechanical stimulus [3], disrupted calcium homeostasis [4], reduced hematopoiesis [5], and altered bone metabolism [6]. To help preserve bone during spaceflight, resistance training, nutrition, and antiresorptive drugs have been studied as countermeasures [7,8,9,10,11,12]. Upon returning to Earth, astronauts may never recover their pre-spaceflight bone health [1,13,14], putting them at risk for early-onset osteoporosis.

Changes to bone structure are the result of bone adaptation, consisting of bone modeling and remodeling [15]. Bone modeling generally occurs during skeletal development and fracture healing, and it tends to increase bone’s mass and strength [16]. In a mature skeleton, bone modeling is likely triggered by increased mechanical strain in the tissue due to increased loading. The tissue responds to this increased load by increasing bone strength to prevent a fracture [17,18,19]. Bone remodeling maintains bone structure through the coupled activity of osteoclasts (bone-resorbing cells) and osteoblasts (bone-forming cells) [20]. These cells are part of a temporary anatomic structure called the basic multicellular unit (BMU) that regulates bone homeostasis [21]. Rapid bone loss in space may result from an upset in the balance between osteoclast and osteoblast activity, where osteoclasts remove more tissue than osteoblasts restore [22,23].

The precise mechanisms driving bone adaptation and rapid bone loss are unknown, making osteoporosis prevention and treatment challenging for both space travelers and individuals on Earth. Being able to estimate changes in bone structure would help researchers understand the mechanisms of bone adaptation, test theories against experimental in vivo data, and plan long-duration spaceflight. Several computational methods exist to simulate bone adaptation [24,25,26,27,28,29]. Early computer simulations were limited to synthetic data and could not be validated with in vivo observations [24,25]. With advances in high-resolution computed tomography (CT) imaging, in vivo bone structures were used to simulate more realistic bone adaptation [26,27,29]. Notably, Müller developed an algorithm called simulated bone atrophy (SIBA) that modeled age-related bone remodeling from micro-CT images of bone tissue [27]. Schulte and colleagues developed a more general simulation based on mechanics that was validated with an in vivo mouse model [28].

With all these techniques, the final bone structure is determined from the initial bone structure and bone adaptation parameters alone (i.e., rate of bone adaptation). An initial bone structure may be accurately acquired from in vivo high-resolution CT imaging [27,29]; however, determining accurate and participant-specific model parameters is challenging. Typically, model parameters are selected from previous experimental studies [26,27,29] or by optimizing a median subject [28]. These techniques are appropriate for animal studies in controlled experiments where genetics, diet, and exercise can be assumed to be the same, but may not be appropriate for in vivo human studies where individual differences in bone adaptation are likely.

Recently, we developed a technique to solve for participant-specific bone adaptation parameters from serial high-resolution CT images of changing bone microarchitecture [30]. We demonstrated the technique on multiple serial synthetic datasets and a single case of longitudinal in vivo data. Our current approach employs a model where bone adaptation depends on surface advection (i.e., scaling constant) and the local mean curvature of the trabecular structure, which was previously shown to mimic age-related bone loss [27,29]. Other models of bone adaptation models (e.g., load-driven) could be explored in the future using the same framework but solving for different relevant patient-specific model parameters. In the present study, we tested our model using a cohort of astronauts as an accelerated case of age-related bone loss and recovery.

We hypothesized that bone adaptation during and after spaceflight are dependent on surface advection and local trabecular curvature. We used the previously developed technique to identify participant-specific bone adaptation parameters, to predict short- (~six months) and long-term (~12 months) bone adaptation, and to verify that bone adaptation during spaceflight and recovery can be modeled via mean curvature and advection.

## 2. Methods

### 2.1. Participants and HR-pQCT Image Acquisition

Participants are from the TBone study, which investigated the effects of long-duration spaceflight (approximately six months) on bone quality [31,32]. Seventeen astronauts were recruited from the National Aeronautics and Space Administration (NASA), Canadian Space Agency (CSA), European Space Agency (ESA), and Japan Aerospace Exploration Agency (JAXA). They were selected for 3.5- to 7-month missions to the international space station (ISS). Crewmembers included 14 men and 3 women with a mean age 46.9 years (SD 6.7), height 177.7 cm (6.0), body mass 79.1 kg (7.7), and body mass index 25.0 kg/m^2^ (2.1). One participant did not complete all measurements and was excluded, leaving 16 participants in the study. Participants gave written informed consent, and the study was approved by the University of Calgary Conjoint Health Research Ethics Board (REB14-0573), NASA Institutional Review Board (NASA7116301606HR), ESA Human Research Multilateral Review Board, and JAXA International Review Board for Human Research.

One of four medical radiation technologists acquired high-resolution peripheral quantitative computed tomography (HR-pQCT, XtremeCT II, Scanco Medical, Brüttisellen, Switzerland) scans of the dominant and non-dominant distal tibia using a standard in vivo scanning protocol [33]. Scans had an isotropic voxel size of 61 μm and volume of interest of 168 slices over a 10.2 mm length. The resulting three-dimensional images had edge lengths of approximately 600 × 600 × 168 voxels. We acquired tibia scans 22.5 mm proximal from the reference line. All scans were graded for motion artifact and had scores less than or equal to 2 [34].

HR-pQCT measurements were taken before spaceflight launch (L), within two weeks of the astronauts’ return from spaceflight (R+0), and six (R+6) and 12 (R+12) months after their return. The technicians independently placed the reference line for baseline (L) scans at the plateau portion of the tibial endplate. For follow-up scans (R+0, R+6, and R+12), technicians placed the reference line as close as possible to the baseline reference line.

We used the manufacturer’s semi-automated contouring method [35] to generate masks of the cortical and trabecular compartments (Image Processing Language, v5.16, Scanco Medical AG, Brüttisellen, Switzerland). The whole-bone and trabecular masks were defined from the periosteal and endosteal surfaces, respectively. The cortical mask was defined as the difference between the whole-bone and trabecular masks.

### 2.2. Image Pre-Processing

Three-dimensional image registration was used to align the greyscale follow-up to baseline images for each participant (SimpleITK, v1.2.4, Insight Software Consortium). We implemented an intensity-based, rigid body registration technique similar to our previous work [36] using Mattes mutual information similarity metric [37] and Powell optimization. The registrations were initialized with a translation aligning the intensity-based centers of mass. Follow-up images were transformed to the baseline image and resampled using a 3rd-order basis-splines interpolator [38]; masks were transformed and resampled with a nearest neighbor interpolator.

Once the images were aligned in image space, we determined the largest common volume of interest (VOI) as the union of the masks from all registered scans. Our analysis used only the trabecular bone compartment, which was isolated based on the trabecular VOI. Finally, we applied a Gaussian filter (sigma 0.8, support 1.0), fixed threshold defined by the manufacturer for the trabecular compartment (320 $mg\;HA/cm^{3}$), and largest connected components filter to binarize the images.

### 2.3. Computational Technique

Previously, we described a forward technique to simulate bone adaptation from a single high-resolution CT image and bone adaptation parameters [29], and an inverse technique to identify participant-specific bone adaptation parameters from serial high-resolution CT images [30]. The forward solver uses the level set equation and embedded image data ϕ to implicitly track the bone surface [39,40]:

(1a) $$\phi_{t}+F\left|\nabla \phi \right|=0,$$

(1b) $$\phi \left(\overset{⃑}{x},t=0\right)=\phi_{0},$$

where the subscript *t* denotes the partial time derivative, $\left|\nabla \right|$ denotes the magnitude gradient, F is the speed function, and $\phi_{0}$ is an initial image from which future images are simulated. We calculated $\phi_{0}$ from binary images using the signed distance transform [41]. F is the speed at which the trabecular bone surface is changing. Previously, we suggested that the speed is a function of the local mean curvature of the trabeculae [29,30]:

(2) F=a−bκ

The parameter a is the advection constant, which implicitly captures factors including genetics, hormones, and mechanics. Advection is transport by bulk motion and is a scaling factor in the overall rate of bone adaptation. The term bκ represents the local mean curvature and captures geometric changes as hypothesized in [27]. The parameter κ (mean curvature) may be directly calculated from the embedding images ϕ [40]. The level set equation is solved numerically using forward Euler time discretization, an upwind scheme for advection and a second-order difference for the curvature term [29]. The aim of this work is not to test the forward nor inverse solvers, which were previously demonstrated and validated [29,30]. Rather, this work aims to test the curvature-driven model (Equation (2)) in vivo.

**Figure 1:**
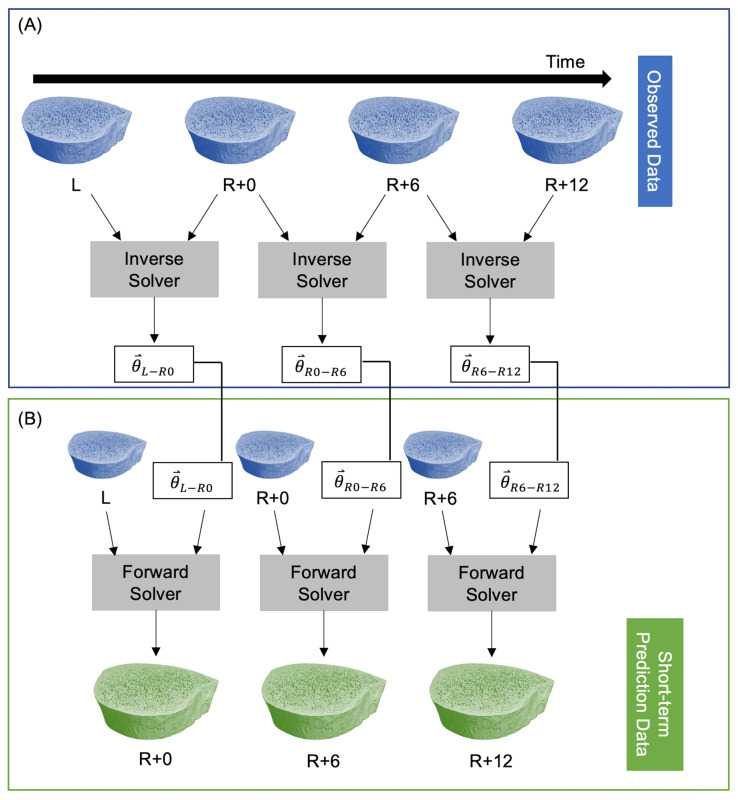
Procedure for predicting short-term participant-specific bone adaptation. (**A**) The observed data are fed into the inverse solver to estimate model parameters at each time interval. (**B**) A single measurement and the corresponding model parameters are fed into the forward solver to predict bone adaptation.

The inverse solver determines the model parameters $\overset{⃑}{\theta }=\{a,b\}$ from two sequential images of changing bone microarchitecture. This is akin to a linear regression where model parameters $\beta_{i}$ in a linear model are fit to observed data. In the current study, model parameters are $\overset{⃑}{\theta }=\{a,b\}$, the model is the speed function in Equation (2), and observed data are longitudinal HR-pQCT images. The inverse solver [30] minimizes a cost function that estimates the difference between the non-overlapping regions of the predicted and observed bone volumes using a linear least squares initialization and Adam gradient descent [42]. The inverse solver’s inputs are two high-resolution CT scans and the time between measurements; the solver returns the model parameters $\overset{⃑}{\theta }=\{a,b\}$ for that time interval. After determining a set of model parameters for each participant with the inverse solver, those participant-specific parameters can be inserted into our model of bone adaptation to simulate future microarchitectures using the forward solver. To predict short-term bone adaptation, we used the inverse solver to estimate participant-specific model parameters at all three time intervals: L to R+0 (${\overset{⃑}{\theta }}_{L-R0}$), R+0 to R+6 (${\overset{⃑}{\theta }}_{R0-R6}$), and R+6 to R+12 (${\overset{⃑}{\theta }}_{R6-R12}$). Next, we used the forward solver to predict bone adaptation for each participant using their unique bone modeling parameters for each time interval. For both the forward and inverse solvers, we used the mission duration in days for L to R+0, and the number of days between measurements for R+0 to R+6 and R+6 to R+12. This resulted in predicted bone volumes corresponding to the R+0, R+6, and R+12 measurements (Figure 1).

**Figure 2:**
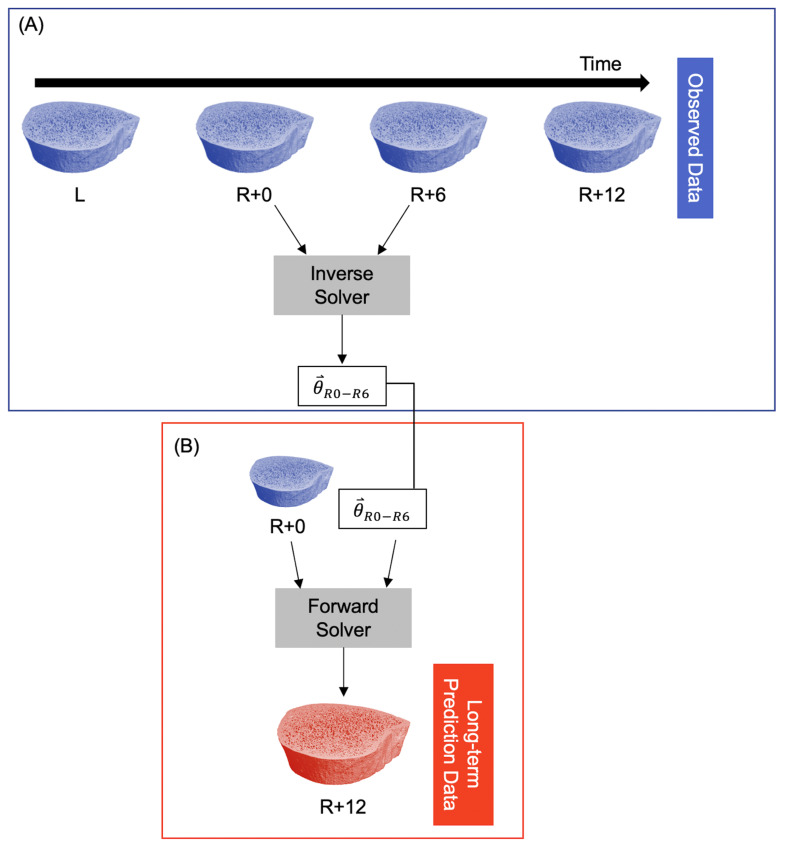
Procedure for predicting long-term participant-specific bone adaptation (12 months). (**A**) The observed data are fed into the inverse solver to estimate model parameters for the time interval R+0 to R+6. This is identical to the procedure in Figure 1A. (**B**) The R+0 observed data and model parameters are fed into the forward solver to predict the bone structure at R+12.

To predict long-term bone adaptation (i.e., >6 months), we used the model parameters for the first six months of recovery (${\overset{⃑}{\theta }}_{R0-R6}$) to extrapolate what their microarchitecture might look like at 12 months of recovery using the exact number of days between the R+0 and R+12 scans. This resulted in a predicted bone volume corresponding to the R+12 measurement (Figure 2).

Previously, we described the two hyper-parameters that control the optimization algorithm: the learning rate for gradient descent (η) and the gradient scaling factor (γ) [30]. We selected η = 5 × 10^−5^ and γ = 5 × 10^−4^ from a sensitivity analysis for this specific astronaut population. Convergence for the inverse solver was defined as the L2 norm $|{\overset{⃑}{\theta }}^{k+1}-{\overset{⃑}{\theta }}^{k}|<$ 1 × 10^−8^ where k is the iteration number in gradient descent. We noted that simulations that exceeded 4000 iterations would likely never converge and were excluded.

### 2.4. Morphometry and Surface Metrics

To compare short- and long-term predictions with observations, we calculated static and dynamic morphometric outcomes as well as embedding metrics. Static morphometric outcomes include trabecular bone volume fraction (Tb.BV/TV; %), thickness (Tb.Th; mm), separation (Tb.Sp; mm), and number (Tb.N, $mm^{-1}$), which were calculated with the manufacturer’s software (Image Processing language, V5.42, Scanco Medical, Brüttisellen, Switzerland).

Local sites of bone formation and resorption were identified by overlaying images from two time points [43]. Registered binary images from subsequent measurements were added to one another, resulting in a three-valued image. Voxels only present in the first image were considered resorbed bone, voxels only present in the second image were considered formed bone, and voxels present in both images were considered quiescent [43,44,45]. The three-valued images served as input for three-dimensional rendering [43,45] and dynamic bone morphometry calculations [45].

Comparing observed and predicted dynamic bone morphometry indicates whether the prediction matched observed rates of bone adaptation. Bone formation rate (BFR, %/day), bone resorption rate (BRR, %/day), mineral apposition rate (MAR, μm/day), and mineral resorption rate (MRR, μm/day) are based on the volume of formed or resorbed bone, which were extracted from the three-valued images [45]. BFR is based on counting voxels that represent the amount of formed bone volume per original bone volume per day; BRR is calculated analogously with resorbed bone volume. MAR (MRR) is defined using the sphere-fitting morphological method to measure the thickness of formed (resorbed) bone volume divided by the number of days between measurements [45]. Formed and resorbed bone thicknesses were calculated using the same algorithm as for trabecular thickness [46].

Mineralizing surface (MS, %) and eroding surface (ES, %) are based on the surface of formed and resorbed bone. Formed and resorbed surfaces were extracted from the three-valued images by multiplying the bone surface voxels by formed and resorbed bone volumes, respectively. Thus, MS is the percentage of formed bone surface per total bone surface, and eroding surface (ES, %) is the percentage of eroded surface per total bone surface.

Embedding metrics compare predicted and observed bone volumes and were defined previously [30]. The Dice coefficient measures the volume of intersection over the total volumes [47], and the symmetric distance measures the distance between two surfaces. These metrics evaluate both the solver convergence and model accuracy. Previously, we found that predictions with a symmetric distance greater than the isotropic voxel size were not accurate [30]. So, in this study, we rejected predictions with symmetric distances greater than 61 μm. In the non-rejected predictions, embedding metrics determined how well the model fits the data.

### 2.5. Statistical Analysis

We assessed whether trabecular bone adaptation during spaceflight and recovery depends on the local mean curvature of the trabeculae by studying the model parameters as well as by comparing static and dynamic morphometry between predicted and observed bone adaptation. We also calculated embedding metrics which compare predicted and observed bone volumes. All statistical analyses were performed (R, v1.0.153), and F-tests determined statistical significance (*p* < 0.05).

We studied the effect of time and anatomical site on the advection and curvature constants with a linear mixed effects model. The linear mixed effects model included fixed effects of time (R+0, R+6, and R+12), site (right and left tibia), and the interaction between time and site. Each participant had a random effect intercept.

Linear regression analysis, Bland–Altman plots [48], and a linear mixed effects model compared the short-term (i.e., 6-month) prediction and observed morphometry at all measurements. The linear mixed effects model included fixed effects of time (R+0, R+6, and R+12), group (observed and predicted), and the interaction between time and group. Each participant had a random effect intercept. Linear regression analysis, Bland–Altman plots, and a mixed effects model compared observed, short-term prediction, and long-term prediction static morphometry at R+12. The linear mixed effects model included fixed effects of group (observation, short-term prediction, and long-term prediction) and a random intercept for each participant. We did not compare short- and long-term dynamic morphometry, as these measures are based on different time intervals. Therefore, a paired Student’s *t*-test compared long-term prediction and observed dynamic morphometry at R+12.

The Dice coefficient and symmetric distance were calculated as previously described [30]. A linear mixed effects model, with fixed effect of time and random intercepts, determined the effect of time on embedding metrics in the short-term prediction. A paired Student’s *t*-test determined the effect of group (short- and long-term prediction) on embedding metrics at R+12.

## 3. Results

### 3.1. Short-Term Prediction

Of the 96 datapoints in the short-term prediction (16 participants × 2 sites × 3 time intervals), 22 did not converge within 4000 iterations. Of the remaining 74 datapoints, four were rejected as the symmetric distance was greater than 61 μm. This resulted in 70 datapoints for the remainder of the analysis. The predicted parameters varied between individuals and showed marked differences between the in-flight and recovery phases that were explored.

**Figure 3:**
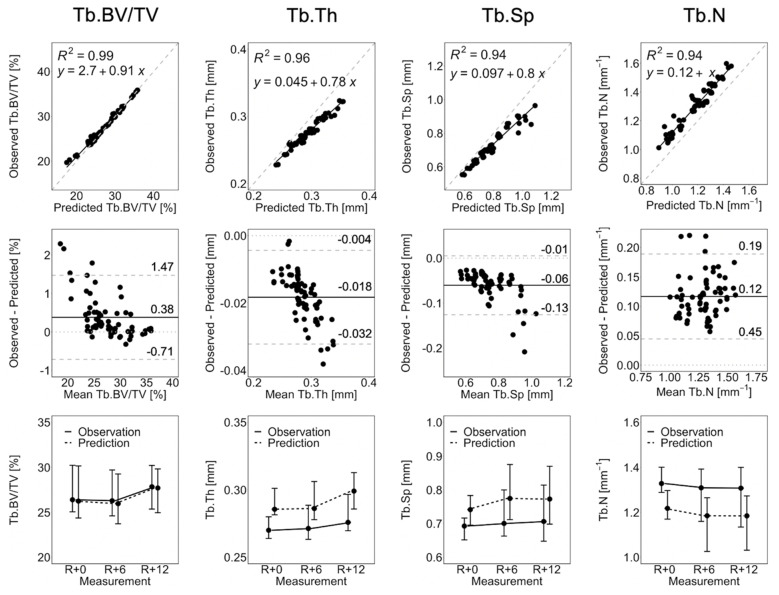
Linear regression, Bland–Altman, and line plots for Tb.BV/TV, Tb.Th, Tb.Sp, and Tb.N comparing the observation and short-term prediction for the 70 converged short-term prediction datapoints. In the regression plots (**top row**), the grey dashed line denotes the line of unity, and the black solid line denotes the regression line. In the Bland–Altman plots (**middle row**), the grey dashed lines denote the limits of agreement, the black solid line denotes the mean, and the grey dotted line denotes zero error. The line plots (**bottom row**) show the median and interquartile range of all participants and anatomical sites (i.e., right and left tibia).

Figure 3 shows the linear regression, Bland–Altman plots, and line plots comparing observed and short-term static morphometry. Table 1 summarizes the results of the linear mixed effects model. Observed Tb.BV/TV is closely matched by the prediction, as indicated by *R*^2^ = 0.99, a linear regression slope close to 1.0, and close agreement in the line plots (Figure 3), as well as mean percentage errors of less than 2.5% at all time points (Table 1). This is confirmed by the linear mixed effects model, which shows no significant difference between observed and predicted Tb.BV/TV (Table 1). Despite being highly correlated (*R*^2^ > 0.94), the observed Tb.Th, Tb.Sp, and Tb.N are not accurate. From the Bland–Altman plots in Figure 3, the prediction over-estimates Tb.Th and Tb.Sp, with large values of Tb.Th and Tb.Sp having larger errors. Tb.N is underestimated by the prediction. The resulting mean percentage errors (6.2 to 9.4%, Table 1) are significant for the linear mixed effects model. There is no significant effect of time indicating that static morphometrics do not change significantly between measurements. There is a significant group effect but no group-by-time interactions, meaning that observations and predictions are significantly different at all measurements.

Observed BFR, BRR, MS, and ES are not matched by the prediction. The linear mixed effects model indicates that the prediction underestimates BFR, BRR, MS, and ES (*p* < 0.0001, Table 2) at all measurement intervals, resulting in large mean percentage errors (59.2 to 81.8%). We observed some significant effects of time and group-by-time interactions. BFR is significantly lower (*p* < 0.01) at the last time interval than the first two intervals in observed data only. BRR and ES are significantly higher (*p* < 0.01) during spaceflight than in the first and second recovery periods in both observed and predicted data. MS is significantly lower during spaceflight than the first six months of recovery in both observed and predicted data (*p* < 0.01). These results indicate an elevated rate of bone loss and gain during spaceflight and recovery, respectively.

MAR and MRR are well matched by the curvature-driven model. From the linear mixed effects model, there are no significant differences between observed and predicted MAR nor MRR (Table 2), and mean percentage errors are less than 6.0%. MAR and MRR are significantly lower during the first and second six months of recovery than during spaceflight (*p* < 0.05) in both observed and predicted data. There are no group-by-time interactions.

Table 3 reports the median (Q1, Q3) embedding metrics comparing observation and short-term prediction, thus, measuring the fit of the curvature-driven adaptation model to the astronaut population. These metrics indicate good local agreement between the model and observed data. There are no significant effects of time, indicating that the Dice coefficient and symmetric distance are similar at all measurements.

### 3.2. Long-Term Prediction

Of the 32 long-term prediction datapoints (16 participants × 2 sites), four did not converge in 4000 iterations. For the long-term prediction, we used symmetric distance to measure optimization and model accuracy. The optimization quality (i.e., convergence) is measured at R+6, whereas the model accuracy is measured at R+12. Recall the R+6 measurement was used to determine the bone adaptation parameters, so poor symmetric distance at R+6 likely indicates poor convergence. We excluded two datapoints with symmetric distance greater than 61 μm at R+6, resulting in 26 long-term prediction datapoints. Six of these datapoints did not converge for the short-term prediction at R+12 and were excluded when comparing short- and long-term static morphometry.

**Figure 4:**
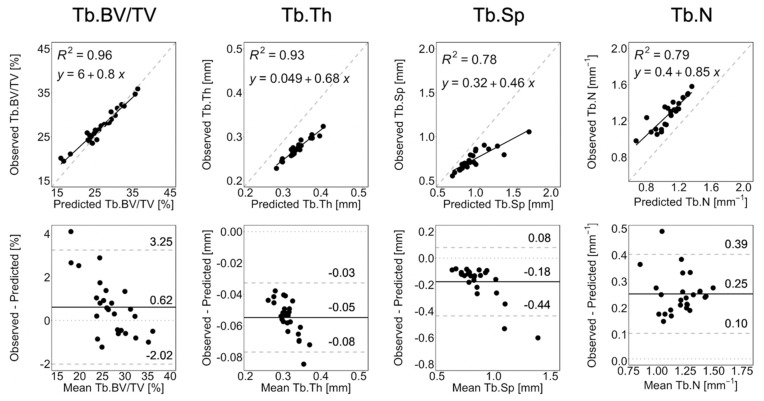
Linear regression and Bland–Altman plots for Tb.BV/TV, Tb.Th, Tb.Sp, and Tb.N comparing observation and long-term prediction for all 26 converged long-term prediction datapoints at R+12. In the regression plots, the grey dashed line denotes the line of unity, and the black solid line denotes the regression line. In the Bland–Altman plots, the grey dashed lines denote the limits of agreement, the black solid line denotes the mean, and the grey dotted line denotes zero error.

**Figure 5:**
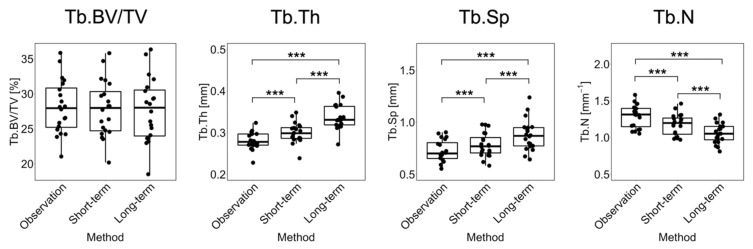
Boxplots for Tb.BV/TV, Tb.Th, Tb.Sp, and Tb.N comparing observation, short-term, and long-term predictions for the 20 common datapoints at R+12. Significance is determined from F-tests: *** = *p* < 0.0001.

Figure 4 shows the linear regression and Bland–Altman plots comparing observed and long-term static morphometry, Figure 5 shows boxplots of static morphometry, and Table 4 summarizes the results of the linear mixed effects model at R+12. Observed and predicted Tb.BV/TV are highly correlated (*R*^2^ = 0.96, Figure 4) and not significantly different (*p* > 0.05, Figure 5), but the Bland–Altman plots show obvious proportional bias (Figure 4). Short- and long-term Tb.BV/TV are also similar (*p* > 0.05, Figure 5). Although observed and long-term Tb.Th, Tb.Sp, and Tb.N are highly correlated (*R*^2^ > 0.94, Figure 4), they are significantly different (*p* < 0.0001, Figure 5), with mean percentage errors ranging from 18.1 to 20.6%, indicating that we did not accurately predict these parameters in the long-term. Tb.Th, Tb.Sp, and Tb.N determined at R+12 in the short- and long-term predictions are significantly different (*p* < 0.0001, Figure 5).

BFR, BRR, MS, and ES are not accurately predicted in the long-term, as indicated by significant differences (*p* < 0.0001, Table 4) and large mean percentage errors (23.4 to 34.6%) between observation and prediction. MAR and MRR are significantly different (*p* < 0.05, Table 4) but have a small mean percentage error (3.7 to 4.6%).

The median (Q1, Q3) Dice coefficient and symmetric distance compare the observation and long-term prediction at R+12. These scores are 0.85 (0.84, 0.86) and 47.3 (46.3, 61.6), respectively. The short-term prediction has a significantly higher Dice coefficient and symmetric distance than the long-term prediction (*p* < 0.0001), indicating that the short-term prediction better matched the observed data.

**Figure 6:**
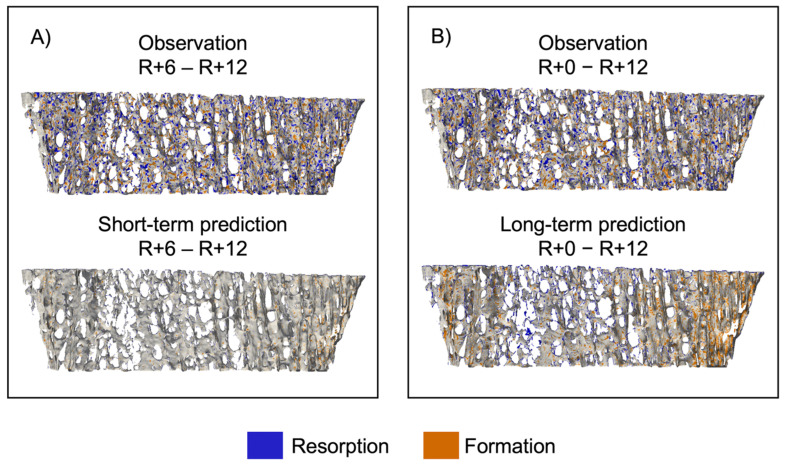
Spatial patterns of bone formation and resorption represented as an overlay between baseline and follow-up images of a representative dataset. (**A**) Comparison of observation and short-term prediction and (**B**) comparison of observation and long-term prediction. Voxels only present in the first image were considered resorbed bone (blue), voxels only present in the second image were considered formed bone (orange), and voxels present in both images were considered quiescent (grey).

Figure 6 shows sites of bone formation and resorption for a representative participant for both the short- and long-term prediction experiments [45]. In the short-term experiment (Figure 6A), the observed bone volume has multiple sites of bone formation and resorption, but the predicted bone volume has few sites of either formation or resorption. For the long-term experiment (Figure 6B), in the predicted volume, we see formation around the perimeter and resorption in the center, indicating that the curvature-driven model preferentially forms and resorbs tissue in regions of dense and sparse trabeculae, respectively.

## 4. Discussion

This study estimated participant-specific bone adaptation during and after spaceflight from HR pQCT images of changing bone microarchitecture. We modeled bone adaptation as an interface-evolution problem where bone adapts as a function of surface advection and the trabeculae’s local mean curvature. By comparing static and dynamic morphometry, as well as local embedding metrics between the modeled and observed data, we determined that the curvature-driven bone adaptation model predicted some of the microstructural bone changes observed during spaceflight and recovery. Although the model did not predict all the parameters, we have established a framework to test this and other future models in vivo on a participant-specific basis in any population.

Studies have attempted to predict bone adaptation from high-resolution CT images [26,27,28] because of the interest in testing mechanobiological theories and predicting future bone health. For example, Schulte and colleagues developed a simulation of ovariectomy-induced and load-induced bone adaptation in animal models, and they used a median subject to determine model parameters for the population [28]. In the ovariectomy model, they found no significant differences between the observed and predicted static morphometry, but they found significant differences between the observed and predicted dynamic morphometry. This is a similar trend to the findings in our study. However, their technique for determining model parameters may only apply to animal studies with minimal genetic variability and controlled activity. In human studies, bone adaptation is highly variable, so subject-specific models are likely necessary. We tuned the estimation to each individual in a population using computational techniques rather than assuming a group-mean adaptation.

Our findings suggest that care must be taken when deciding the time frame for predictions. In general, the short-term predictions matched the observations better than the long-term predictions, likely because small errors in the model result in amplified errors when projected over a long time frame. Another possibility is that there may be a change in the mechanism of recovery after the first six months that does not extrapolate to the second six months. Bone remodeling markers regain pre-flight levels within six months of recovery but decline to lower than pre-flight values between six and 12 months [1]. So, our assumption that the model parameters would not change between six and 12 months of recovery is likely not correct, which is consistent with our previous experimental findings of transitioning biomarkers in the year post-flight [32]. Prior information about bone turnover biomarkers may indicate how far into the future a model can predict bone adaptation. Although challenging to develop, a new bone adaptation model could include terms that account for changes in biomarkers or exercise to capture the most important drivers of bone adaptation during spaceflight.

In general, the observed and predicted static morphometry agreed well. In fact, differences between the predictions and observations for static morphometric outcomes were within the least significant change (LSC) of the scanner [49,50]. At the tibia, HR-pQCT LSC was 0.52%, 0.006 mm, 0.044 mm, and 0.10 $mm^{-1}$ for Tb.BV/TV, Tb.Th, Tb.Sp, and Tb.N, respectively [36]. In the short-term prediction, we found mean absolute errors between the modeled and observed Tb.BV/TV, Tb.Th, Tb.Sp, and Tb.N of 0.58%, 0.02 mm, 0.07 mm, and 0.12 $mm^{-1}$, respectively. Despite this strong agreement in global morphometry, we noticed visual differences in the observed and predicted bone structures. Likely, local errors in static morphometry were averaged out when calculating global metrics, giving a reasonable result. The Dice coefficient and symmetric distance were better at distinguishing non-concordant bone surfaces despite having a similar averaging effect to global static morphometry. Dynamic morphometry, particularly the bone formation (resorption) rate and mineralizing (eroding) surface, gave the best indication of discrepancies in bone surfaces and model fit. This is an important finding, as it indicates that two bones may adapt at different rates and have poor local agreement but still have similar morphometric outcomes. Future work should focus on developing models that predict dynamic morphometry and local changes, as these measures best indicate a model’s accuracy.

The observed and predicted static morphometry were likely correlated because the morphometry inherent to the observed bone structures was carried through to the predicted structures. Despite being correlated, Tb.Th and Tb.Sp were overestimated, and Tb.N was underestimated by the curvature-driven model. Since the mean curvature flow preferentially erodes thinner sections, thin trabeculae likely eroded more quickly in the predicted data than in the observed data. If these trabeculae were lost, it would explain the increase in average Tb.Th and Tb.Sp, and the decrease in Tb.N that was observed. This explanation was qualitatively confirmed by observing bone formation and resorption sites, where we noticed that very small trabecular structures were resorbed.

The curvature-driven model accurately captured mineral apposition and resorption rates, which are two key parameters for studying bone turnover. However, the curvature-driven model underestimated the bone formation (resorption) rate and mineralizing (eroding) surface. Moreover, the visualized predicted surfaces showed minimal sites of bone turnover, indicating that the model made few changes in the bone structure. This discrepancy in dynamic morphometry is explained by the magnitude of the advection and curvature constants, which gave median bone adaptation rates of −96.2, −88.6, and −86.9 μm/year for L - R+0, R+0 - R+6, and R+6 - R+12, respectively, for an average trabecular thickness of 265.0 ± 28.3 μm [51]. So, in a six-month period, the modeled surface likely changed by less than a voxel. The long-term prediction showed more sites of bone adaptation because, over 12 months, the surface changes were measurable (i.e., greater than one voxel). Additionally, when computing the advection and curvature constants, the optimization minimizes the difference in volume between the modeled and observed data; the optimization is not concerned with the rate or spatial location of bone adaptation. Future work could investigate how different cost functions in the optimization improve both static and dynamic morphometric outcomes simultaneously.

The advection and mean curvature model modifies the bone surface through principles of interface evolution [29], which is appropriate given that bone changes only occur on the bone surface. However, our findings imply that this model does not capture all the physiological and mechanical factors driving adaptation. Fortunately, the inverse technique is versatile. So, performing a similar experiment with a different bone adaptation model in the future (such as the mechanostat or other geometric flows) or applying the model to different populations (such as post-menopausal women) is feasible. Given its generalizability, this work provides a method to test bone adaptation models with a variety of in vivo cohorts and could become a basis to advance our knowledge of bone adaptation mechanisms.

A limitation of this study was that certain simulations did not converge at all time intervals or anatomical sites (i.e., left or right tibia). In vivo data in particular may suffer from poor convergence since we previously demonstrated that the solver was accurate and converged in synthetic data [30]. The solver may not have converged if noise (i.e., motion artifact) was present, if the participant had low bone mineral density (i.e., sparse data in osteoporotic participants) [30], or if the solver converged to a local minimum. In this study, we found that a motion artifact could negatively affect convergence; nine scans had a motion score of 2, 77% of which were in simulations that did not converge. But several scans with motion scores of 1 were also in simulations that did not converge, meaning other factors are at play. Low bone mineral density was unlikely to be a problem since the astronaut population had good initial bone health overall [31]. Most likely, the remaining simulations that did not meet the convergence criteria converged to a local minimum.

Another limitation of this work was the small population size, which is often the case in human space studies, but it was further reduced by simulations that did not converge. The small sample affects our ability to study convergence and model accuracy in the astronaut population. We were also constrained by the computational time as each simulation took ~2 weeks at a high-performance computing center (Advanced Research Computing, University of Calgary), but the speed could be greatly improved by parallelizing the inverse solver. Finally, the HR-pQCT image resolution is limiting when studying local bone adaptation. Given an average trabecular thickness of 265.0 ± 28.3 μm [51], an error of one voxel (61 μm) results in an almost 25% error in local trabecular thickness. Such an error may be negligible when calculating gross trabecular thickness, but this error may be non-negligible and likely affects the prediction quality when studying local bone adaptation.

Future work should consider some of these limitations. For example, it could improve the efficiency of the solver so that it is feasible to study larger populations, possibly by implementing parallelization. Convergence may be improved by testing other algorithms suited to the non-convex problem we are solving. We would also like to consider substituting other models into the solver to test other geometric flow or strain-based models that might be better at capturing local changes. Lastly, the ability to predict long-term changes may be improved by incorporating longitudinal data representative of the dominant mechanism of change. In our data, it is possible that the first six months of recovery represent a distinct phase immediately after landing that is not representative of the long-term exposure to normal gravity on Earth.

In conclusion, we modeled participant-specific bone adaptation in vivo during and after spaceflight by solving an inverse interface evolution problem. We implemented a curvature-driven model, where the bone adaptation rate is a function of surface advection and the local mean curvature of the trabeculae. Although the curvature-driven model had prediction errors in this cohort, the inverse technique is promising and could test other bone adaptation models in vivo. Validating bone adaptation models has implications both clinically and academically, as researchers can identify the mechanisms and drivers of bone loss to further understand the determinants of bone health. In the special case of spaceflight, predicting future bone loss may provide important insight into long-duration missions, such as future missions planned to Mars.

## Figures and Tables

**Table 1:** Percent error (%) in static morphometry between observation and short-term prediction at each measurement reported as mean ± standard deviation.

Parameter	Error R+0 [%]	Error R+6 [%]	Error R+12 [%]	*p*-Value
Tb.BV/TV	1.6 ± 2.0	2.5 ± 3.2	1.0 ± 1.3	0.393
Tb.Th	6.2 ± 2.6	6.2 ± 1.8	7.2 ± 2.3	<0.0001
Tb.Sp	7.4 ± 2.1	9.1 ± 5.2	8.2 ± 3.0	<0.0001
Tb.N	8.6 ± 2.3	9.4 ± 3.6	8.9 ± 2.1	<0.0001

Tb.BV/TV = trabecular bone volume fraction, Tb.Th = trabecular thickness, Tb.Sp = trabecular separation, Tb.N = trabecular number, R+0 = return plus zero months, R+6 = return plus six months, R+12 = return plus 12 months. *p*-values less than 0.05 are considered significant and denote the main effect of group (observation vs. short-term prediction). *p*-values were calculated from F-tests with the linear mixed effects models. Although errors are reported in this table, the linear mixed effects model used absolute values of static morphometry. There are no significant effects of time and no group-by-time interactions.

**Table 2:** Observed and predicted (short-term) dynamic morphometry at each measurement interval reported as median (Q1, Q3).

	L - R+0		R+0 - R+6		R+6 - R+12		
Parameter	Observation	Short-Term Prediction	Observation	Short-Term Prediction	Observation	Short-Term Prediction	*p*-Value
BFR [%/day]	0.12 (0.11, 0.16)	0.04 (0.02, 0.04)	0.12 (0.12, 0.14)	0.04 (0.03, 0.04)	0.11 (0.10, 0.13)	0.04 (0.03, 0.04)	<0.0001
BRR [%/day]	0.13 (0.12, 0.17)	0.05 (0.04, 0.07)	0.12 (0.11, 0.13)	0.04 (0.03, 0.05)	0.11 (0.10, 0.13)	0.04 (0.03, 0.05)	<0.0001
MS [%]	25.5 (24.29, 27.90)	5.73 (4.42, 6.32)	27.82 (25.83, 32.60)	6.36 (5.77, 7.60)	27.09 (25.12, 28.60)	6.23 (5.60, 7.26)	<0.0001
ES [%]	28.13 (26.41, 30.94)	9.27 (8.33, 12.12)	26.49 (25.26, 29.24)	7.49 (6.58, 10.55)	26.32 (25.59, 28.24)	8.18 (6.87, 9.52)	<0.0001
MAR [μm/day]	0.73 (0.69, 0.92)	0.72 (0.66, 0.88)	0.75 (0.71, 0.77)	0.73 (0.67, 0.75)	0.71 (0.66, 0.74)	0.67 (0.64, 0.73)	0.477
MRR [μm/day]	0.73 (0.70, 0.93)	0.73 (0.67, 0.89)	0.74 (0.71. 0.77)	0.74 (0.69, 0.76)	0.69 (0.66, 0.74)	0.68 (0.64, 0.73)	0.162

BFR = bone formation rate, BRR = bone resorption rate, MS = mineralizing surface, ES = eroding surface, MAR = mineral apposition rate, MRR = mineral resorption rate, L - R+0 = measurement interval from launch to return, R+0 - R+6 = measurement interval from return plus zero months to return plus six months, R+6 - R+12 = measurement interval from return plus six months to return plus 12 months. *p*-values less than 0.05 are considered significant and denote the main effect of group (observation vs. short-term prediction). *p*-values were calculated from F-tests with the linear mixed effects models. There are some significant effects of time and some group-by-time interactions, which are indicated in text.

**Table 3:** Embedding metrics reported as median (Q1, Q3) comparing observation and short-term prediction.

	R+0	R+6	R+12
Dice coefficient	0.86 (0.84, 0.88)	0.87 (0.85, 0.88)	0.87 (0.86, 0.88)
Symmetric distance [μm]	41.6 (38.6, 46.2)	40.6 (37.5, 48.4)	40.0 (37.5, 43.2)

R+6 = return plus six months, R+12 = return plus 12 months.

**Table 4:** Observed and predicted (long-term) dynamic morphometry at the R+12 - R+0 measurement interval reported as median (Q1, Q3).

Parameter	Observation	Long-Term Prediction	*p*-Value
BFR [%/day]	0.06 (0.06, 0.07)	0.04 (0.04, 0.05)	<0.0001
BRR [%/day]	0.06 (0.05, 0.06)	0.04 (0.03, 0.05)	0.0008
MS [%]	28.15 (27.06, 33.62)	18.74 (17.11, 21.17)	<0.0001
ES [%]	26.32 (25.48, 28.56)	20.35 (17.20, 25.11)	0.0004
MAR [μm/day]	0.35 (0.34, 0.38)	0.35 (0.33, 0.35)	0.0174
MRR [μm/day]	0.35 (0.34, 0.37)	0.35 (0.33, 0.35)	0.0174

BFR = bone formation rate, BRR = bone resorption rate, MS = mineralizing surface, ES = eroding surface, MAR = mineral apposition rate, MRR = mineral resorption rate, R+0 - R+12 = measurement interval from return plus zero months to return plus 12 months. All 26 converged long-term prediction datapoints were included in this analysis. *p*-values less than 0.05 are considered significant and denote the difference between groups (observation vs. long-term prediction). *p*-values were determined using a paired Student’s *t*-test.

## Data Availability

No data are available.
